# Microsystems for Cell Cultures

**DOI:** 10.3390/bios12040190

**Published:** 2022-03-23

**Authors:** Iordania Constantinou

**Affiliations:** 1 Institute for Microtechnology, Technische Universität Braunschweig, 38124 Braunschweig, Germany; i.constantinou@tu-braunschweig.de; 2 Zentrum für Pharmaverfahrenstechnik (PVZ), Technische Universität Braunschweig, 38106 Braunschweig, Germany

Microfabricated systems are increasingly being utilized in biotechnological, biomedical, and pharmaceutical research and development as replacements for traditional in vitro cell cultures, bioreactors, and animal experiments (Figure 1). Such microsystems include microfluidics for cell culture, comprising microphysiological systems (MPS), also known as organ-on-a-chip (OoC) platforms, and microbioreactors.

OoCs are designed to recapitulate the complex biochemical and biophysical conditions of living cells and tissues and replicate their natural microenvironment. The dynamic control over the conditions cells are exposed to is facilitated by active and passive components, such as microvalves, as well as by integrated microsensors. The ability to in situ control and monitor both the cell culture microenvironment and the biological responses of the cultured cells allows for elevated data relevance and sets microfluidic platforms apart from traditionally used cell culture tools. In addition to OoC platforms, microbioreactors have become versatile bioprocess engineering tools used for microbial and mammalian cell cultivation. Such cell culture technologies are often equipped with miniaturized sensors that provide feedback, allow process monitoring and control, and ensure the necessary cell culture conditions are maintained.

This Special Issue aims to assemble a collection of novel methods and microsystems for cell cultivation. Advances in the areas of system design, sensor integration, as well as target applications are welcome. Two original articles have already been published in this Special Issue. Stelzle et al. reported a parallelizable microfluidic platform with a multi-well plate format, where ten independent cell culture chambers support the modelling of cellular barriers co-cultured with 3D tumor spheroids. Real-time barrier function was assessed using integrated electrodes for transepithelial/transendothelial electrical resistance (TEER) measurements [1]. Grünberger et al. have contributed a protocol paper on how to successfully perform a microfluidic cultivation experiment with the aim to promote broader applicability of microfluidic cell culture devices in the field of life science and promote their ongoing advancement [2]. The focus of the published protocol lies in the successful and reproducible cultivation of cells (bacteria, algae, fungi, mammalian cells) in microfluidic systems. An overview of the most frequently occurring challenges and pitfalls is provided, along with guidelines on how to overcome them.

Further contributions in the form of original work, reviews or protocols on the broad topic of microsystems for cell cultivation are welcome.

## Figures and Tables

**Figure 1:**
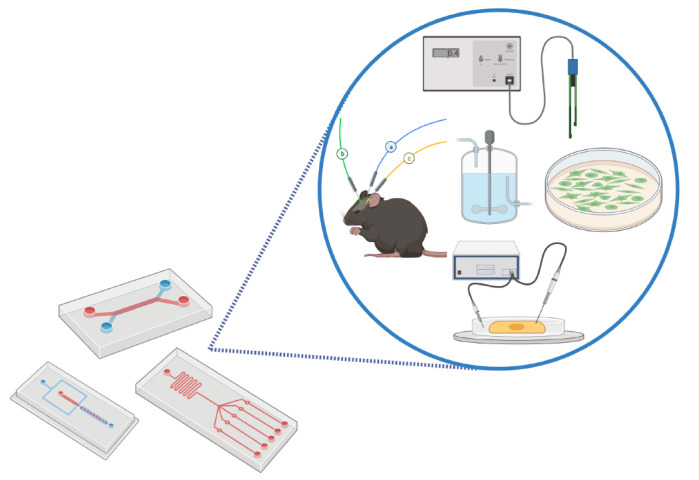
Microfabricated platforms, often equipped with integrated sensors, are increasingly utilized as replacements for traditional in vitro cell cultures, bioreactors, and animal experiments. Figure contributed by Christian Sieben, Helmholtz Centre for Infection Research. Created with BioRender.com (accessed date 21 March 2022).
